# Vasoactive intestinal peptide inhibits TNF-α-induced apoptotic events in acinar cells from nonobese diabetic mice submandibular glands

**DOI:** 10.1186/ar2671

**Published:** 2009-04-08

**Authors:** Mario Calafat, Luciana Larocca, Valeria Roca, Vanesa Hauk, Nicolás Pregi, Alcira Nesse, Claudia Pérez Leirós

**Affiliations:** 1 Departamento de Química Biológica, Facultad de Ciencias Exactas y Naturales, Universidad de Buenos Aires, Ciudad Universitaria, Pabellón II, 4° piso, 1428, Buenos Aires, Argentina; 2 Consejo Nacional de Investigaciones Científicas y Técnicas, Avda. Rivadavia 1917, CP C1033AAJ, Buenos Aires, Argentina

## Abstract

**Introduction:**

The role of apoptotic secretory epithelium as a pro-inflammatory triggering factor of exocrine dysfunction is currently explored in Sjogren's syndrome patients and in the nonobese diabetic (NOD) mouse model. Vasoactive intestinal peptide (VIP) has anti-inflammatory effects in various models of chronic inflammation. Our goal was to analyse the effect of TNF-α on apoptotic mediators in isolated acinar cells from NOD submandibular gland and their modulation by VIP.

**Methods:**

Acinar cells were isolated from submandibular glands of 16-week-old NOD females with salivary flow decline. Age-matched BALB/c females or eight-week-old NOD females were used as controls. Apoptotic mediators and TNF-α receptor expression were assessed by immunoblotting and RT-PCR, caspase 3 activity was assessed by optical density at 405 nm with Ac-DEVD-pNA as a substrate and chromatin condensation by Hoechst stain. They were evaluated in resting conditions and after a 3.5 or 6 hours of culture with TNF-α. VIP effects in acinar cells were assessed at 100 nM in TNF-α-treated cultures and VIP receptor functional assays by radio immunoassay (cAMP) or enzymatic detection (amylase).

**Results:**

NOD acinar cells at 16 weeks present an increased expression of TNF-α receptor1 together with increased Bax, tumour protein 53-induced nuclear protein1α (TP53INP1α), caspase 3 activity and chromatin condensation. Acini from NOD mice were more sensitive to TNF-α-induced increases of apoptotic mediators than control cells. VIP inhibited TNF-α-induced apoptotic events through functional VPAC1 receptors coupled to the protein kinase A (PKA) signalling pathway.

**Conclusions:**

Our results indicate that acinar cells isolated from submandibular glands of NOD mice with salivary dysfunction are more sensitive to apoptosis induced by TNF-α which could be prevented by VIP through a PKA-mediated pathway.

## Introduction

Sjögren's syndrome is an autoimmune rheumatic disease characterised by a progressive loss of exocrine secretion that affects women in a 9:1 ratio [1-3]. The pathogenic mechanisms of disease are presently unknown and the active involvement of epithelial acinar cells producing inflammatory mediators has been discussed previously [4]. Hence, studies on acinar cell cultures from patients with Sjögren's syndrome and biopsies indicate an aberrant expression and activation of inflammatory mediators together with defective activity of key enzymes involved in saliva secretion [4-6].

Observations that acinar cells may be actively involved in the pathogenesis of Sjögren's syndrome thus encourage the search for molecules that could mediate these processes, which could even arise as biomarkers for diagnosis and/or disease activity. Evidence obtained with the nonobese diabetic (NOD) Sjögren's mouse model also supports this view providing a feasible approach for this search. The NOD mouse model of Sjögren's syndrome, at the pre-diabetic stage, has the unique characteristic of developing a deep secretory dysfunction with mild infiltration of the glands [7] consistent with a structural-dysfunctional aetiology.

The role of apoptosis of secretory epithelium as a triggering factor of early dysfunction and autoimmune response has been explored in patients with Sjögren's syndrome and in the pre-diabetic NOD mouse model. Apoptosis evaluated by terminal UTP nucleotide end-labelling method (TUNEL) and Fas/FasL-mediated apoptosis have already been reported in both cases [5,8-10]. In keeping with this, we have shown early signalling alterations in submandibular glands from NOD mice involving neural isoform of nitric oxide synthase (NOS 1), calcium-calmodulin kinase II and cGMP [11,12]. Moreover, with a lower activity of NOS 1 in exocrine glands and higher serum levels of TNF-α, we recently reported an increased DNA fragmentation with increased Bax expression in isolated acinar cells from NOD submandibular glands at the Sjögren's syndrome-like period [13,14]. TNF-α and TNF-α receptors (TNF-αR) are important cell surface inducers of apoptosis leading to proteolysis and DNA fragmentation [15]. The ability of TNF-α to regulate apoptosis in isolated pancreatic acini and experimental pancreatitis has previously been reported [16].

Vasoactive intestinal peptide (VIP) is a neuroimmunopeptide with several actions on exocrine glands. In addition to inducing vasodilation and exocrine secretion, VIP was postulated as a trophic factor for acini [17] and it has shown strong anti-inflammatory properties in several models of chronic autoimmune and immune-mediated inflammatory diseases [18-20]. Regarding NOD mice at the Sjögren's syndrome-like stage, treatment with VIP *in vivo *from the fourth week of age reduced Th1 cytokine levels in the serum and increased IL-10 [21]. In line with this, VIP was proposed as one of the promising approaches for the treatment of Sjögren's syndrome based on VIP gene-transfer experiments in NOD females [3,22].

The aim of the present study was to analyse apoptotic events involving TNF-α/TNF-αR in isolated acini from NOD mice submandibular glands and to explore the ability of VIP to modulate these effects. In addition to Bax and other pro-apoptotic and anti-apoptotic signals, we investigated the expression of two proteins encoded by tumour protein 53-induced nuclear protein 1 gene (*TP53INP1)*, TP53INP1α and β whose over-expression was associated to Bax expression and apoptosis [23,24]. Our present data indicate that isolated acini from submandibular glands of NOD mice present increased chromatin condensation, TNF-αR, Bax and TP53INP1α expression, and caspase 3 activity than normal BALB/c mice. TNF-α was more potent in inducing pro-apoptotic mediators and nuclear chromatin condensation in NOD acinar cells. VIP inhibited TNF-α-induced apoptotic events through a cAMP-mediated pathway.

## Materials and methods

### Animals

NOD and BALB/c female mice were bred and maintained in the Central Animal Care Facility at the School of Exact and Natural Sciences, University of Buenos Aires. Mice were fasted overnight with water *ad libitum *before used. They were routinely tested for blood glucose levels (Wiener Laboratory, Rosario, Argentina) and considered pre-diabetic as their values of serum glucose on two occasions over a 24-hour period did not significantly differ from those of control mice (0.9 ± 0.1 g/l, n = 27). NOD mice of 16 weeks of age used throughout this study presented a reduced saliva flow rate (about 40% reduction) as compared with BALB/c control mice. All studies were conducted according to standard protocols of the Animal Care and Use Committee of the School of Exact and Natural Sciences, University of Buenos Aires, Argentina.

### Submandibular acinar cell isolation and treatments

Submandibular glands were quickly removed and immediately transferred to ice-cold RPMI 1640 10% fetal bovine serum (FBS) (Gibco, Invitrogen, Carlsbad, CA, USA). Acinar cell isolation was performed as previously described [25]. For each experiment, the tissue coming from about 10 NOD and 10 BALB/c submandibular glands (pooled for each mice group) was minced into small fragments and digested in 2.5 ml RPMI containing Collagenase IV (Sigma, St Louis, MO, USA) (100 U/ml), 10% FBS and 0.1 g/L soybean trypsin inhibitor at 37°C in a shaking water bath for 10 minutes (120 cycles/minute), dispersed with a plastic pipette, filtered through a nylon mesh (150 meshes).

The acinar cells were centrifuged at 400 g for 10 seconds for three times in fresh RPMI medium containing 10% FBS and were seeded on flat-bottom 24-well microtitre plates (Corning Glass, Corning, NY, USA) and incubated for two hours at 37°C in a humidified incubator with 5% carbon dioxide to separate glandular immune adherent cells. The purified suspension presented a homogenous population of acinar cells with a minimal presence (less than 5%) of mononuclear immune cells as revealed by flow cytometry analysis. Viability of acinar cell suspension was stated by acridine orange/propidium iodide staining and trypan blue exclusion [26]. Resulting acini were plated and cultured in RPMI 1640 10% FBS for the times indicated for each determination. When used, recombinant TNF-α (Promega, Madison, WI, USA) (5 and 10 ng/ml) was added to acinar cells for 3.5 hours for RT-PCR experiments or six hours for nuclear condensation, caspase 3 activity and immunoblotting experiments. In some experiments, cells were pre-incubated for 30 minutes with 100 nM VIP (Neosystem, Strasbourg, France) before TNF-α addition in the presence or absence of H89 (1 μM).

### Nuclear chromatin condensation

Cells were fixed with 4% paraformaldehyde in PBS (v/v) for 20 minutes at 4°C, exposed to 0.05 g/l Hoechst 33258 dye in PBS for 30 minutes at room temperature, washed three times with PBS and, finally, mounted in 50% glycerol in PBS (v/v), as previously described [27]. Fluorescent nuclei with apoptotic characteristics were detected by microscopy under ultraviolet illumination at 365 nm. The images (400×) were photographed with a Nikon Coolpix 5000 (Nikon, Tokyo, Japan) and digitalised. For differential cell counting at least 500 cells were analysed.

### Immunoblotting

Acinar cells were homogenised at 4°C in 50 mM Tris-HCl buffer at pH 7.5 with 0.15% Triton X-100 (SIGMA, St Louis, MO, USA) and protease inhibitors as previously reported [12]. Once centrifuged at 5000 g for 10 minutes at 4°C, supernatants were frozen at -80°C until used and an aliquot of each sample was separated for protein determination. Extracts (50 μg protein/lane) were subjected to 10% SDS-PAGE, transferred onto nitrocellulose membranes and immunoblotted with rabbit polyclonal anti-Bax (N-20, Santa Cruz Biotechnology, Inc., Santa Cruz, CA, USA), rat monoclonal anti-TP53INP1 (E-12) or goat polyclonal anti-actin (Santa Cruz Biotechnology, Inc., Santa Cruz, CA, USA) used as primary antibodies. Membranes were incubated overnight and revealed with peroxidase-conjugated secondary antibodies (1:3000) followed by enhanced chemiluminescence detection system (ECL; Pierce, Rockford, IL, USA). Densitometric analysis of protein levels was performed with ImageQuant software (GE Healthcare, Chalfont St Giles, UK).

### RNA extraction and PCR amplification of cDNA

Total RNA was extracted from acinar cells with Trizol (Gibco, Carlsbad, CA, USA) as described [14]. Reverse-transcribed cDNAs were amplified using specific primers for Bax, TP53INP1s, TNFR1 and BclxL with glyceraldehyde 3-phosphate dehydrogenase (GAPDH) primers serving as an internal control. GAPDH is considered an appropriate housekeeping gene in this model [8]. 5'-Bax: 5'-GGAATTCCAAGAAGCTGAGCGAGTGT-3' and 3'-Bax: 5'-GGAATTCTTCTTCCA GATGGTGAGCGAG-3' were used as forward and reverse primers, respectively. The reaction yielded a cDNA fragment 394 base pairs (bp) in length [28]. For TP53INP1: forward 5'-GCACCCTTCAGTCTTTTCCTGTT-3' (position 718) and TP53INP1 reverse 5'-GGAGAAAGCAGGAATCACTTGTATC-3' (position 886) [23]. After denaturing for three minutes at 96°C, 30 cycles of amplification using a step program (96°C, 40 seconds; 58°C (for bax), 55°C (for TP53INP1), 30 seconds and 72°C, 1 minute) and a final extension at 72°C 10 minutes was performed.

VPAC1 forward 5'-GTGAAGACCGGCTACACCAT-3' reverse 5'-TGAAGAGGGCCATATCCTTG-3' and VPAC2 forward 5'-CCAAGTCCACACTGCTGCTA-3' reverse 5'-CTCGCCATCTTCTTTTCAG-3'. PCR conditions for VPAC1/VPAC2 were 94°C for 10 minutes, 35 cycles of 94°C for 45 seconds, 55°C for 45 seconds, 72°C for 90 seconds and 72°C for 10 minutes while for Bax were 96°C for 3 minutes, 30 cycles of 58°C for 30 seconds, 72°C for 1 minute and 72°C for 10 minutes. PCR, products were size fractionated on 2% agarose gels and visualised by staining with ethidium bromide using a size molecular marker. Scion Image for Windows program (Scion Corporation, Frederick, MA, USA) for processing. The analysis of images was used to measure areas and the ratio of interest gene vs. housekeeping gene is depicted in each graph.

### Caspase 3 activity

After appropriate treatments, cells were harvested by centrifugation at 1000 g for five minute at 4°C. Cell pellets were washed with 1 ml of PBS, then suspended in 150 μl of lysis buffer (50 mM Tris-HCL, pH 7.4; 1 mM EDTA; 10 mM EGTA; 10 μM digitonina) containing 1 mM phenylmethylsulphonyl fluoride protease inhibitor and maintained for 30 minutes on ice. Cell debris was removed by centrifugation at 15,000 g for 20 minutes at 4°C, 144 μl of cell lysates were transferred to a microplate, 6 μl of 7.8 mM Ac-DEVD-pNA were added and the volume completed to 300 μl with the reaction buffer (100 mM HEPES, pH 7.5, 0.5 mM EDTA, 5 mM DTT, 20% glycerol). Plates were covered and incubated at 37°C for two to six hours until a yellowish colour was observed. The amount of released p-nitroaniline was measured spectrophotometrically at 405 nm in a microplate reader (BioRad, Hercules, CA, USA).

### cAMP levels, amylase activity and secretory profile

Acinar cells were assayed for their functional ability to secrete salivary protein and stimulate cAMP levels in response to VIP by determining basal and VIP-stimulated cAMP by RIA and amylase secretion as previously reported [12,25,29]. Acinar suspension was incubated for 15 minutes in the absence and presence of 100 nM VIP and amylase activity was determined at 30 minutes in the intracellular fraction and in the supernatants (secreted). Percentage of secretion was calculated as the ratio of secretion over total amylase (total amylase is that present in intracellular plus supernatant fractions) and normalised per mg protein. Protein detection was performed using the Micro BCA Protein Assay (Pierce, Rockford, IL, USA) in acinar suspension aliquots.

### Statistical analysis

Statistical significance of differences was determined by the two-tailed t test for independent populations. When multiple comparisons were necessary, the Student-Newman-Keuls test was used after analysis of variance. Differences between groups were considered significant at *P *< 0.05.

## Results

### Apoptosis pattern of acinar cells in resting conditions

**Figure 1:**
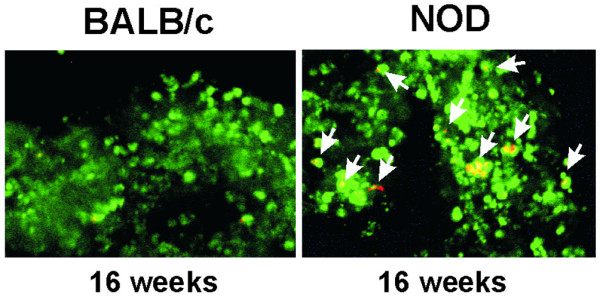
Acinar cell suspension. Acinar cells were stained with acridine orange and propidium iodide (AO/PI). Nuclei of live cells fluoresce green with AO/PI under fluorescence microscopy. Arrows indicate apoptotic cells with yellow-orange fluorescence. These images are representative of five others from similar experiments run with independent non obese diabetic (NOD) and BALB/c acinar samples. Magnification ×200.

**Figure 2:**
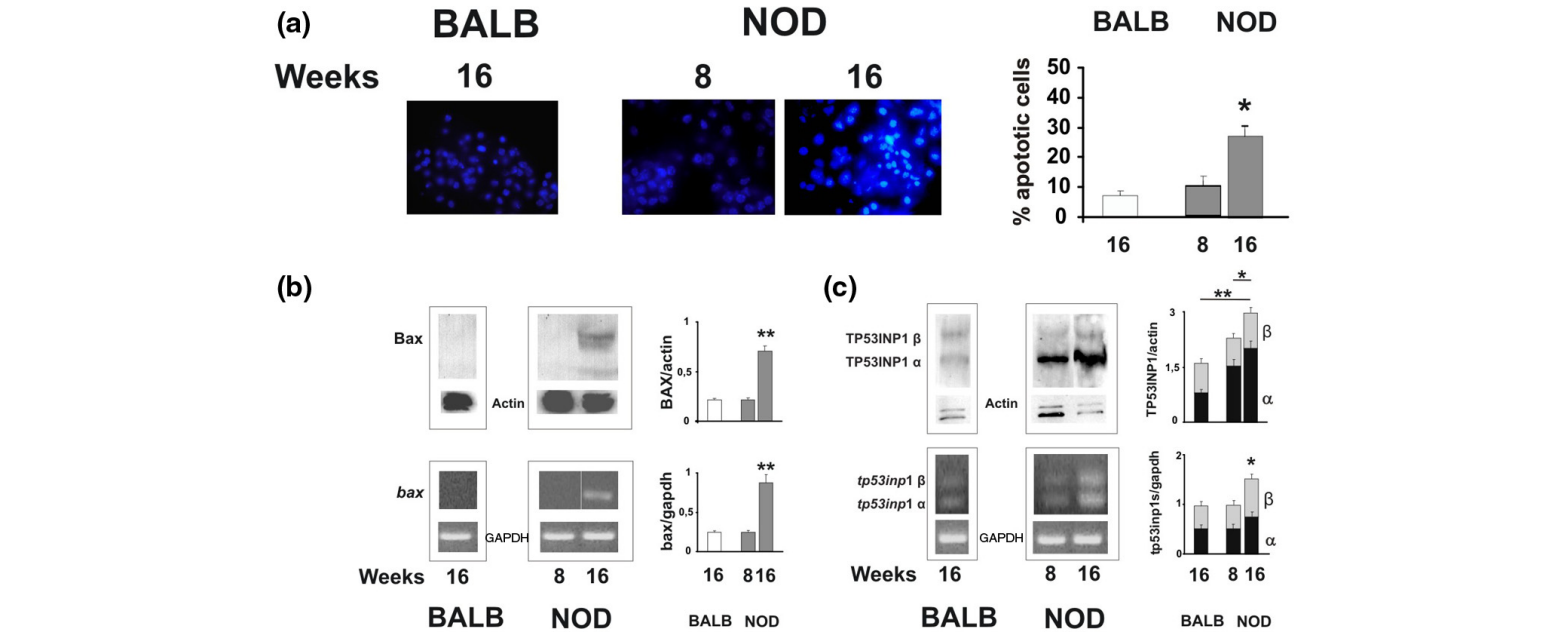
Apoptosis of acinar cells from NOD submandibular glands. **(a) **Chromatin condensation and mRNA and protein expression of **(b) **Bax and **(c) **TP53INP1 were determined in acinar cells from non obese diabetic (NOD) at 16 and 8 weeks of age and 16 week old BALB/c mice. (c) The light grey bar corresponds to TP53INP1β while TP53INP1α is in dark bars. The count of condensed nuclei at 400× after Hoechst staining and RT-PCR or immunoblotting for apoptotic mediator expression were performed as indicated in Material and methods. These images are representative of similar experiments run with independent NOD and BALB/c acinar samples. Results shown on the right side of representative photographs represent the percentage of apoptotic cells as mean ± standard error of the mean (SEM) of at least six experiments. (a) *P < 0.05 vs. BALB/c 16 weeks. The intensity of mRNA or protein bands relative to GAPDH or actin, respectively, were depicted as the mean ± SEM of at least three experiments. (b) *P < 0.05 vs. NOD 8 weeks TP53INP1α; **P < 0.01 vs. BALB/c 16 weeks TP53INP1α and Bax.

Figure 1 shows acinar cell suspension isolated from submandibular glands of NOD and control BALB/c mice, both at 16 weeks of age, stained with acridine orange and propidium iodide. Viable cells fluoresce green under dark field fluorescence microscopy, while nonviable cells fluoresce orange. We investigated further whether freshly isolated acinar cells from NOD mice presented signals of apoptotic events in resting unstimulated conditions (Figure 2). Control acini were obtained from age-matched BALB/c mice and from NOD mice at eight weeks. As shown in Figures 2a and 2b, an increased count of apoptotic acinar cells by Hoechst staining along with an increased expression of Bax at mRNA and protein levels in NOD mice acini compared with control mice was found. An over-expression of TP53INP1 has been associated with Bax expression and apoptosis in acinar cells but not in ductal or Langerhans cells in the pancreas of a mouse model of pancreatitis [24], so we determined TP53INP1 α and β expression in NOD acinar cells. Figure 2c shows TP53INP1 α and β mRNA and protein expression increased only in NOD mice acini compared with BALB/c acinar cells. A faint increase of the TP53INP1α isoform was detected at eight weeks in NOD mice only at the protein level.

### TNF-α-induced apoptosis in NOD acinar cells

**Figure 3:**
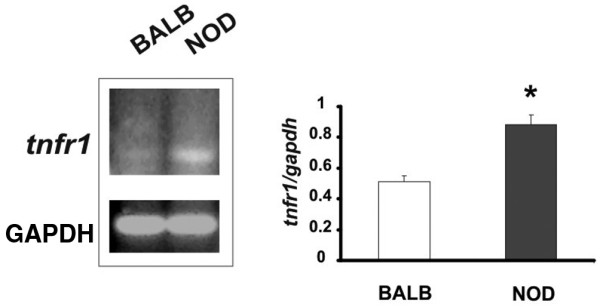
Increased expression of TNF-αR1 in NOD acini. TNF-αR1 mRNA expression in isolated acini from non obese diabetic (NOD) and control mice was determined by RT-PCR as described in Materials and methods. Bands shown are representative of four experiments and results shown for TNF-αR1 band intensity are means ± standard error of the mean of ratios relative to GAPDH for independent NOD (n = 8) and BALB/c (n = 6) samples in experiments run similarly. *P < 0.05 vs BALB/c cells.

With the knowledge that TNF-α/TNF-αR interaction mediates apoptosis in pancreatic acinar cells [16] and the observation that acinar cells isolated from NOD submandibular glands presented several signals of apoptotic events in resting conditions shown above, we first analysed TNF-αR expression in NOD and control isolated acinar cells in basal conditions. Figure 3 shows an increased expression of TNF-αR1 in NOD acini in resting conditions with a negligible expression of TNF-αR2 in both cases (not shown).

**Figure 4:**
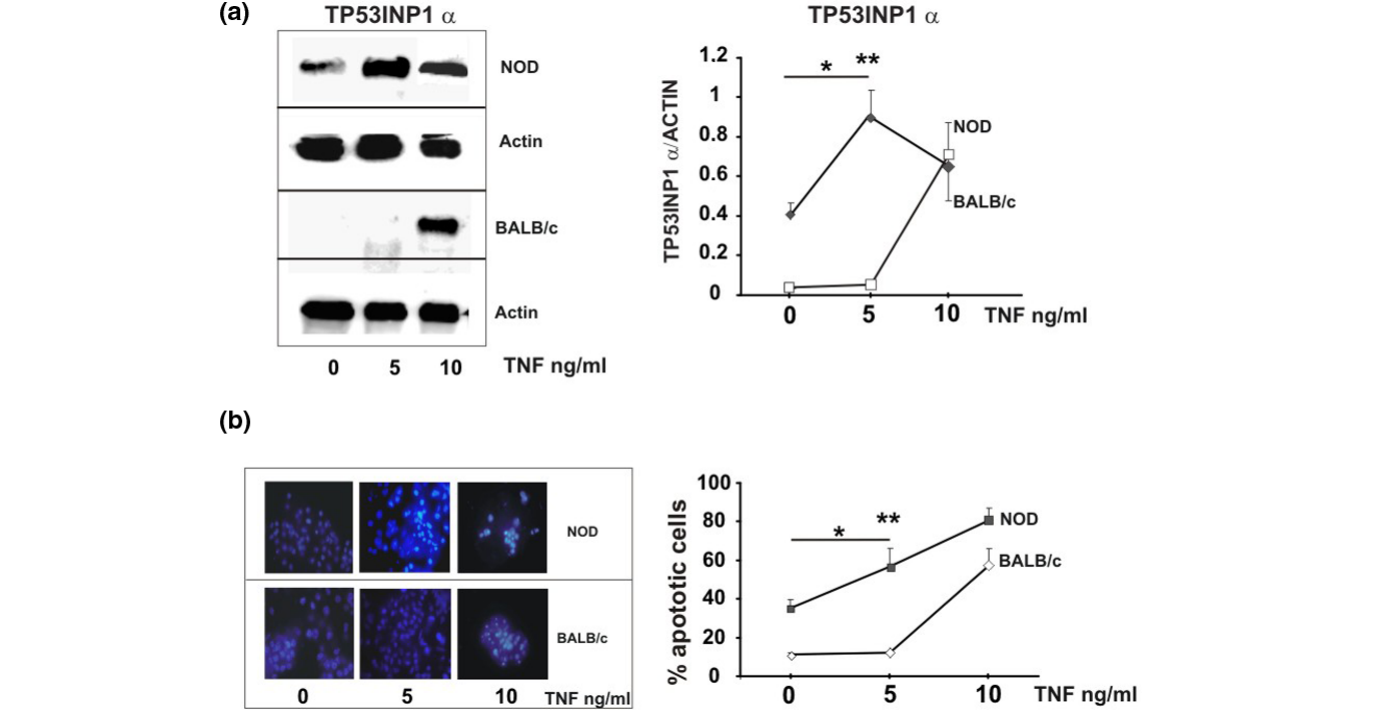
TNF-α-induced chromatin condensation and TP53INP1α expression. The effect of TNF-α (5 and 10 ng/ml) on both BALB/c and non obese diabetic (NOD) isolated acinar cells was determined by **(a) **TP53INP1α expression and **(b) **Hoechst staining as in Figure 1 after six hours incubation of isolated acini with the cytokine at 37°C with 5% carbon dioxide. Gel bands and apoptotic acini shown are representative of separate experiments with NOD and control cells. Results on percentage of apoptotic cells and TP53INP1 expression are means ± standard error of the mean of five and three experiments respectively. *P < 0.05 vs. basal values of NOD acini; **P < 0.01 vs 5 ng/ml TNF in BALB/c cells.

With the aim of investigating the ability of TNF-α to induce apoptosis in acinar cells from NOD and control mice submandibular glands, we then determined the effect of TNF-α at 5 and 10 ng/ml. Figures 4a and 4b show that TNF-α induced TP53INP1α expression at 5 ng/ml only in NOD acini and this effect was paralleled by an increased condensation of nuclear chromatin. At a higher concentration (10 ng/ml), the cytokine could reproduce these effects on normal acinar cells (Figure 4).

**Figure 5:**
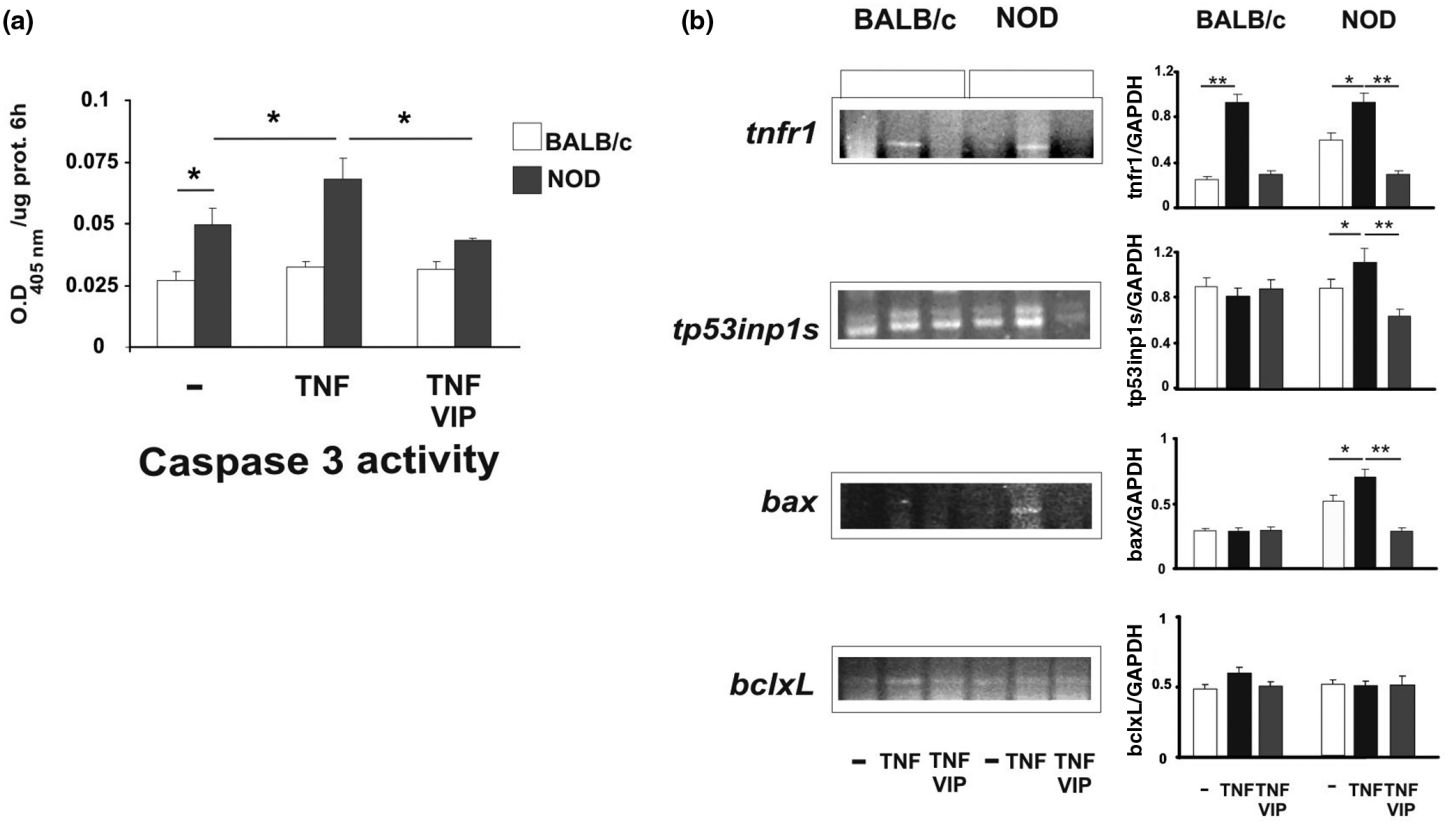
VIP inhibition of TNF-α-induced apoptotic events. **(a) **Caspase 3 activity, TNF-αR1, **(b) **TP53INP1s, Bax and BclxL mRNA expression were determined after TNF-α (5 ng/ml) incubation as in Figure 2 in the presence or absence of 100 nM vasoactive intestinal peptide (VIP) as described in Materials and methods. Results of caspase activity are means ± standard error of the mean (SEM) of at least four experiments run independently. The intensity of the bands for TNF-αR1, TP53INP1s, Bax and BclxL relative to GAPDH are shown as means ± SEM ratios from at least three experiments run independently with non obese diabetic (NOD) (n = 12) and BALB/c (n = 10) samples. *P < 0.05; **P < 0.01.

At the concentration that TNF-α induced chromatin condensation and TP53INP1 expression only in NOD acini, it also increased caspase 3 activity, and increased expression of Bax, TP53INP1 and its own receptor TNF-αR1, but it did not modify the expression of BclxL (Figure 5). To analyse whether VIP could prevent or modulate this effect of TNF-α, we performed the same experiments in the presence or absence of the neuropeptide.

### Inhibitory effect of VIP on TNF-α-induced apoptosis

**Figure 6:**
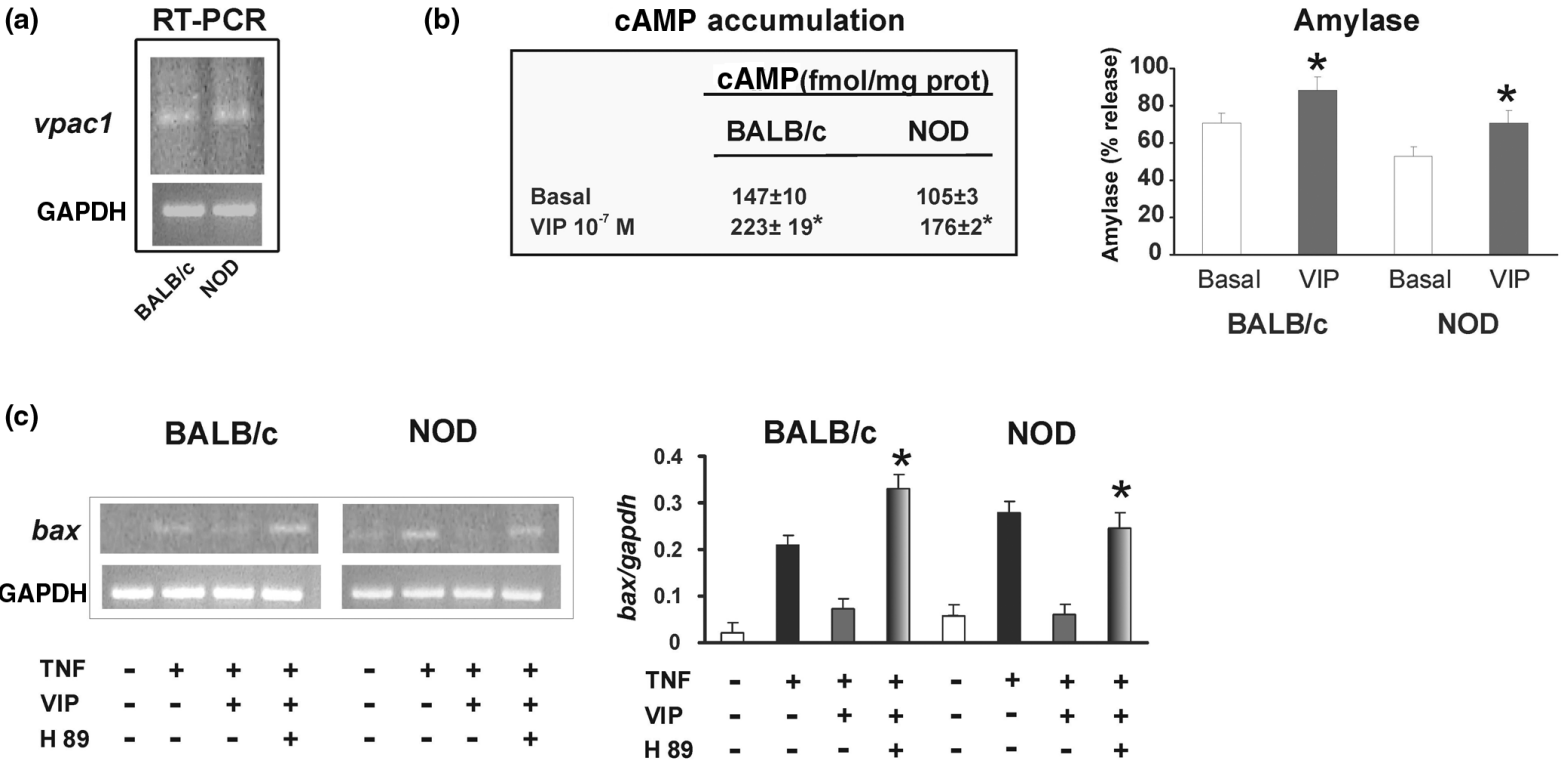
VIP receptor expression in acinar cells and their activation through PKA. **(a) **The levels of VPAC1 receptor mRNA expression were determined in total RNA extracted from isolated acinar cells of both strains of mice as described in Material and methods. **(b) **Cells were incubated in the presence or absence of 100 nM vasoactive intestinal peptide (VIP) for 15 minutes for amylase secretion assays and cAMP accumulation. cAMP was determined by radioimmunoassay and amylase by means of an enzymatic assay as described in Material and methods. *P < 0.05 vs. basal. **(c) **Bax mRNA expression levels were determined after TNF-α (20 ng/ml) incubation as in Figure 2 in the presence or absence of 100 nM VIP and pre-incubated or not with 1 μM H-89 (15 minutes). The intensity of the bands for Bax relative to GAPDH was measured as in Figure 5 and expressed as means ± standard error of the mean for at least three experiments run independently with non obese diabetic (NOD) (n = 12) and BALB/c (n = 10) samples. *P < 0.05 vs. TNF+VIP.

We first studied the effect of VIP on apoptotic mediators induced by TNF-α. The preincubation of acinar cells with 100 nM VIP prevented the inducing effect of TNF-α on all of the apoptotic events shown (Figure 5). As it is shown in Figures 5a (caspase activity) and 5b (TP53INP1 and bax) TNF-α was unable to induce these factors in control acinar cells although it did increase their expression/activity in the NOD suspension. Moreover, VIP only reduced their expression in NOD acini suggesting that VIP modulation mainly affects TNF-α upregulated factors. To identify and functionally characterise VIP receptors involved in this acinar cell preparation, we investigated VIP receptor expression, cAMP accumulation and amylase secretion. Figure 6a shows that acinar cells express VPAC1 receptors in both strains of mice. In contrast VPAC 2 expression could not be detected at any condition tested (not shown).

Figure 6b shows that VIP receptors on acinar cells are functional because 100 nM VIP stimulated cAMP accumulation and amylase secretion at the same concentrations used for inhibiting apoptotic signals. Note that in basal and VIP-stimulated conditions acinar cells present a lower amylase secretion confirming in this acinar cell preparation the results shown previously in studies of *in vivo *salivary flow stimulation (Figure 6b) [29]. Finally, as expected for a VPAC1-cAMP-PKA-mediated effect, VIP failed to reverse apoptotic signals induced by TNF-α in acinar cells in the presence of PKA inhibitor H89 (Figure 6c). VIP alone did not modify the basal expression of apoptotic mediators or the condensation of chromatin in NOD and control acini (not shown).

## Discussion

We presented evidence to indicate that acinar cells isolated from submandibular glands of NOD mice with salivary dysfunction are more sensitive to TNF-α-induced apoptotic events than BALB/c control cells and that VIP prevents these effects. We also showed that NOD acinar cells are functional for cAMP signalling and amylase secretion through VIP receptors, although they express a number of apoptotic signals and mediators activated in resting conditions that were enhanced with TNF-α and prevented by VIP.

Our conclusions are based on three main observations. First, acinar cells isolated from submandibular glands of NOD mice undergoing salivary flow decline showed increased condensation of nuclear chromatin, caspase 3 activity and Bax, TNF-αR1 and TP53INP1α expression compared with control mice. Second, TNF-α enhanced apoptotic events in NOD acini at a concentration that did not modify various apoptotic mediators in BALB/c acini. However, the treatment of normal BALB/c acinar cells with a higher (10 ng/ml) TNF-α concentration reproduced the apoptotic pattern of resting NOD acini. Third, pretreatment of NOD acini with VIP acting on functional VPAC1 receptors prevented apoptotic events induced by TNF-α.

Apoptosis mediated through Fas/FasL has already been reported in salivary glands of NOD and NOD-*scid *mice with increased Fas expression in both acinar and ductal cells at 18 weeks of age [8]. In contrast, FasL was detected as earlier as eight weeks in NOD submandibular glands [8]. Similarly, caspase 3 gene expression appeared to be increased in submandibular glands of the Sjögren's syndrome-susceptible strain C57BL6/NOD.Aec1Aec2 mice at eight weeks of age but returned to baseline values at the 12th week of age [30]. This supports the notion that early defects in glandular homeostasis leading to apoptotic events might predispose them to the autoimmune response.

Here we showed that acinar cells from NOD mice present a characteristic apoptotic pattern with increased Bax expression, chromatin condensation and caspase 3 activation adding new evidence to previously reported apoptosis markers in NOD whole gland samples [8-10] and DNA fragmentation in NOD acinar suspension [14]. The signs of apoptosis were detected in acinar cells from NOD mice at 16 weeks of age when they had already developed a saliva flow reduction higher than 40% compared with BALB/c mice. Of note, at eight weeks of age NOD females showed normal salivary function [12] and there was no detectable apoptosis of acinar cells, suggesting that apoptotic processes, if present, might be incipient at this early stage and not detectable by the methods used.

Among the pro-apoptotic mediators determined in NOD acinar cells, here we chose to analyse α and β TP53INP1 isoform expression. The overexpression of nuclear TP53INP1s stimulates the transactivation activity on the Bax promoter and induces apoptosis in cell lines and the exocrine pancreas in a model of pancreatitis [23]. Interestingly, immunohistochemical analyses revealed that increased expression of TP53INP1 during the acute phase of pancreatitis was only observed in acinar cells with no staining of Langerhans islets or pancreatic duct cells [24]. Here we showed that NOD acini had an enhanced expression of TP53INP1α in resting conditions from the eighth week of life and when stimulated with TNF-α. The β isoform did not show significant changes as it was also described in mice pancreatitis [23]. The modulation of nuclear proteins Ku70 and Ku80 in a caspase 3-dependent pathway was proved in oxidative stress-induced apoptosis of AR42J pancreatic acinar cells [31]. A sparing action of these partner Ku70 and Ku80 nuclear proteins was suggested, as one subunit may stabilise the other subunit under certain conditions [31].

To our knowledge, this is the first report on an increased TP53INP1 expression in acinar cells from salivary glands in relation to apoptosis, either in the NOD model or in normal mice glands treated with TNF-α. Regarding the NOD model, it is also remarkable that the higher TP53INP1 protein expression observed co-occurred with a lower amylase secretion in functional assays of the acinar cell suspensions from low secretory capacity animals. However, the involvement of TP53INP1α expression previous to overt apoptosis and salivary dysfunction in NOD mice and its putative value as a biomarker for patients with Sjögren's syndrome needs further research.

Our results indicate that an increased expression of TNF-αR and a higher sensitivity to TNF-α underlies the inflammatory/apoptotic profile displayed by acinar cells isolated from submandibular glands of NOD mice in the Sjögren's syndrome-like stage. Increased levels of plasma and saliva TNF-α in NOD mice at the Sjögren's like stage have been reported [13,32]. Interestingly, increased levels of TNF-α in saliva and serum of pre-diabetic NOD mice were shown to correlate with the decline of salivary flow but not with the severity of mononuclear infiltrates measured as focus score and ratio of inflamed area to total glandular area [12,32]. Similarly, compared with age-matched BALB/c mice, we have shown increased bioactive TNF-α produced by peritoneal macrophages of NOD females concomitant with a decline of salivary flow but no signs of severe mononuclear infiltration [33].

Enhanced expression of six of 30 TNF-α superfamily genes was detected at earlier ages in RNA from freshly extracted submandibular glands of C57BL/6.NOD-Aec1Aec2 strain used as a model of Sjögren's syndrome [30]. These results are consistent with previously reported identification of Tnfsf6 (FasL) y Ox40 protein from the TNF superfamily as a potential candidate SjS susceptibility marker [34]. Likewise, evidence that autoantigens fodrin, SS-A (Ro) and SS-B (La), in human salivary gland cells treated with TNF undergo a striking redistribution during apoptosis and relocate to the cell membrane of apoptotic cells has been presented [35]. On the other hand, pancreatic acini produce TNF-α and express TNF-αR1 in a model of pancreatitis suggesting a role of TNF-α in the autocrine regulation of apoptosis [16]. In line with this, NOD acini from submandibular glands showed an increased expression of TNF-αR1 in resting conditions that was enhanced by TNF-α, while several pro-apoptotic mediators but not the anti-apoptotic BclxL were also up-regulated by the cytokine. NOD acini required a lower concentration of TNF-α to promote chromatin condensation, pro-apoptotic mediators and caspase 3 activation than BALB/c acini consistent with a higher expression of its own receptor in the former.

Proteomic and genomic approaches in NOD mice and patients samples have allowed the identification of several apoptotic and inflammatory factors as well as acinar cell components as putative biomarkers of Sjögren's syndrome [36-38]. Interestingly, a lower amylase expression in saliva has been proposed as a biomarker in a proteomic approach in patients [36]. However, a correlation between biomarkers, salivary dysfunction and common immunopathological signatures in both NOD mice and patients with Sjögren's syndrome was not found [37,38].

Finally, VIP inhibited TNF-α-induced apoptotic events in NOD acinar cells. VIP has been proved as a potent anti-inflammatory molecule in several models of autoimmune inflammatory disease [18-20]. When given every other day to pre-diabetic NOD females between the 4th and 16th week, it reduced serum Th1 cytokine IL-12 and increased IL-10 [21]. In addition, VIP transfer experiments onto NOD pre-diabetic females reduced the autoimmune response against submandibular glands and reversed salivary flow decline [22]. In line with this, *in vitro *treatment of NOD macrophages with VIP reduced TNF-α, nitric oxide and IL-12 and increased IL-10 produced by peritoneal macrophages [31]. Although VIP has also been shown to inhibit apoptosis through the inhibition of the expression of Fas ligand in activated T lymphocytes [39,40] data are lacking about VIP on TNF-α-mediated apoptosis in immune and non-immune cells. Here we showed that the anti-apoptotic effect of VIP on acinar cells induced with TNF-α involved direct activation of functional amylase secretion-coupled VIP receptors through a PKA-dependent pathway.

## Conclusions

An increased expression of TNF-αR and a higher sensitivity to TNF-α underlies the inflammatory/apoptotic profile displayed by acinar cells isolated from submandibular glands of NOD mice in the Sjögren's syndrome-like stage. The anti-inflammatory neuropeptide VIP was able to inhibit TNF-α-induced apoptotic events through a PKA-mediated pathway. Acinar cells isolated from salivary flow declining glands might serve as a suitable model to analyze combined information about identified biomarkers, cell secretion functional studies and disease severity.

## Abbreviations

bp: base pair; FBS: fetal bovine serum; GAPDH: glyceraldehyde 3-phosphate dehydrogenase; IL: interleukin; NOD: non obese diabetic; NOS: nitric oxide synthase; PBS: phosphate-buffered saline; PKA: protein kinase A; RT-PCR: reverse transcription polymerase chain reaction; TNF-α: tumour necrosis factor-alpha; TNF-α-R: tumour necrosis factor-alpha-receptor; TP53INP1: tumour protein 53-induced nuclear protein 1; TUNEL: terminal UTP nucleotide end labelling method; VIP: vasoactive intestinal peptide.

## Competing interests

The authors declare that they have no competing interests.

## Authors' contributions

MC and CPL designed the study and wrote the manuscript. MC carried out all of the experiments with acinar cells from their isolation. LL helped with fluorescence microscopy and western blot assays. VR and VH participated in caspase 3 activity, salivary flow experiments, glycemia monitoring and NOD colony maintenance. NP and AN helped in data analyses and interpretation of VIP-mediated inhibition of apoptosis signals. CPL supervised the study. All authors read and approved the final manuscript.
